# Case Report: Vogt-Koyanagi-Harada Syndrome Mimicking Acute Angle-Closure Glaucoma in a Patient Infected With Human Immunodeficiency Virus

**DOI:** 10.3389/fmed.2021.752002

**Published:** 2022-01-12

**Authors:** Xue Bai, Rui Hua

**Affiliations:** Department of Ophthalmology, First Hospital of China Medical University, Shenyang, China

**Keywords:** Vogt-Koyanagi-Harada, human immunodeficiency virus, secondary angle-closure glaucoma, choroidal effusion, optical coherence tomography

## Abstract

Vogt-Koyanagi-Harada disease (VKH) is a rare multisystemic inflammatory autoimmune disorder. Glaucoma secondary to VKH frequently occurs during the recurrent phase of anterior uveitis; however, acute angle-closure glaucoma (ACG) secondary to both VKH and human immunodeficiency virus (HIV) infection has rarely been reported. We describe a case of secondary acute ACG involving VKH, characterized by sudden vision loss, moderately elevated intraocular pressure (IOP), shallow anterior chamber, and fully or partially closed angle, in an HIV-infected patient. Both VKH and HIV infection contributed to the occurrence of ACG due to the leakage and forward rotation of the ciliary body, as well as choroidal effusion. The deterioration of IOP and serous macular detachment were observed after initial corticosteroid therapy. Visual acuity and IOP were improved with subretinal fluid absorption after continued corticosteroid therapy. Understanding the response of IOP and serous macular detachment after corticosteroid therapy is important for clinical practice.

## Introduction

Human immunodeficiency virus (HIV) is a lentivirus that infects cells of the human immune system, causing its dysfunction (1). In 2017, an estimated 36.9 million people worldwide had HIV infection, according to the World Health Organization (2). The incidence rate of HIV-related eye diseases has increased correspondingly and is considered one of the most common complications of acquired immune deficiency syndrome. Human immunodeficiency virus-related eye diseases mainly include ocular eye microangiopathy, opportunistic infections, tumors, and immune reconstitution inflammatory syndrome (3). Vogt-Koyanagi-Harada disease (VKH) is a rare multisystemic inflammatory autoimmune disorder characterized by panuveitis, accompanied by neurological and cutaneous manifestations (4). Glaucoma secondary to VKH can be observed at any time after the onset of uveitis, which frequently occurs during the recurrent phase of anterior uveitis, owing to posterior iris adhesion and pupil block, anterior angle occlusion, trabecular meshwork inflammation, inflammatory cells being closed to the trabecular meshwork, and the long-term use of glucocorticoids (5).

Certain patients diagnosed with VKH can develop acute angle-closure glaucoma (ACG), with ACG as the first symptom, including a sudden or gradual increment in intraocular pressure (IOP), shallow anterior chamber, and aqueous flare often accompanied by choroiditis and optic disc oedema (6). However, acute ACG secondary to both VKH and HIV infection has rarely been reported. Herein, we present a case of VKH mimicking acute ACG in an HIV-infected patient and investigate its underlying pathogenesis.

## Case Presentation

**Figure 1:**
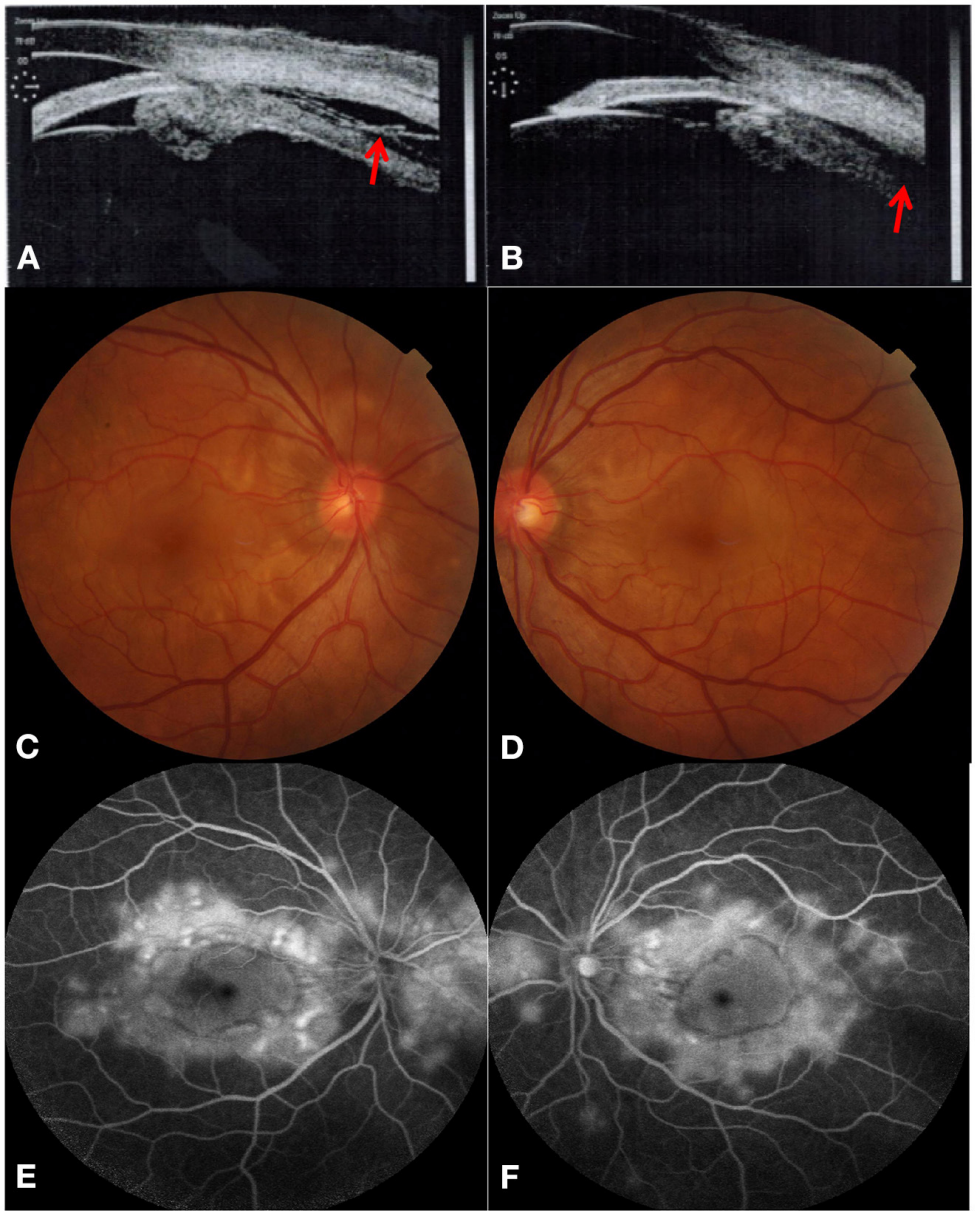
Acute angle-closure glaucoma secondary to Vogt-Koyanagi-Harada disease at initial visit. Ultrasound biomicroscopy revealed both leakage (red arrow) and forward rotation of the ciliary body and secondary angle-closure in bilateral eyes [**(A)** right eye, **(B)** left eye]. Color fundus photograph showed multifocal serous retinal detachments with hyperaemic optic discs in both eyes [**(C)** right eye, **(D)** left eye]. Typical “multiple lake” hyperfluorescence and optic disc leakage are found in the late stage of fundus fluorescein angiography [**(E)** right eye, **(F)** left eye].

A 55-year-old man infected with HIV complained of pain in both eyes, headache, and epicranium ache for 2 days with an episode of fever (38.5°C). In addition, he did not have any systemic symptoms of HIV infection. The patient had normal best-corrected visual acuity (BCVA) for both eyes (the decimal vision for both eyes was 1.0). His symptoms mainly included bilateral conjunctival oedema and superficial scleral hyperaemia without corneal oedema. Shallow peripheral anterior chamber depth (PACD) was evaluated for both eyes on slit-lamp microscopy, which was about one-quarter of the corneal thickness at the 6 o'clock position. In addition, his IOPs were 19 and 21 mmHg in the right and left eye, respectively. Central corneal thickness was 505 mm in the right eye and 494 mm in the left eye. Moreover, ultrasound biomicroscopy (UBM) revealed shallow anterior chambers (central anterior chamber depth of 1.75 mm in the right eye and 1.85 mm in the left eye) and angle closure in both eyes (Figure 1). Therefore, the patient's condition was diagnosed as acute ACG in both eyes.

**Figure 2:**
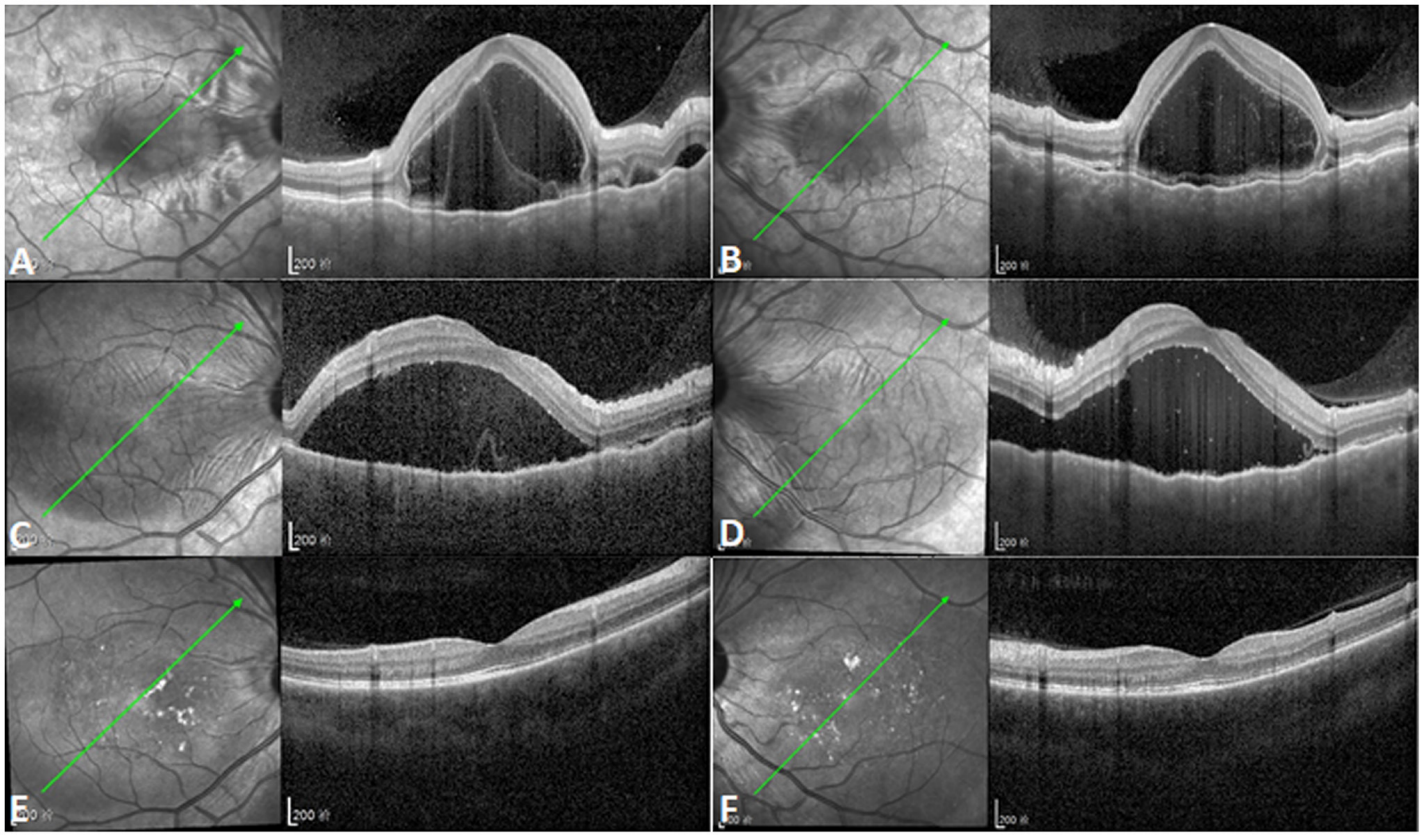
Increased subretinal fluid after initial corticosteroid therapy. **(A,B)** Optical coherence tomography (OCT) showing multifocal serous retinal detachments with choroidal effusion in both eyes (left illustrations indicate the orientation of OCT scanning profile). **(C,D)** Subretinal fluid (SRF) increased and the coalescence of previous multifocal serous retinal detachments was observed in both eyes after initial corticosteroid therapy [left illustrations indicate the orientation of OCT scanning profile, and point-to-point follow-up mode was used for **(A,C)**, **(B,D)**]. **(E,F)** OCT showing complete absorption of SRF with normal macular structure 6 months after the onset of Vogt-Koyanagi-Harada disease [left illustrations indicate the orientation of OCT scanning profile, and point-to-point follow-up mode was used for **(A,E)**, **(B,F)**].

Unfortunately, the patient's BCVA decreased to 0.15 and 0.12 in the right and the left eye, respectively, 1 day later with keratic precipitate (dust type), which indicated acute uveitis. We realized that the previous UBM had already revealed both leakage and forward rotation of the ciliary body. Multifocal serous retinal detachments with hyperaemic optic discs were detected in both eyes through dilated pupils. Moreover, typical “multiple lake” hyperfluorescence and optic disc leakage in the late phase were also observed via fundus fluorescein angiography (Figure 1). In addition, optical coherence tomography (OCT) revealed multifocal serous retinal detachments with choroidal effusion in both eyes (Figure 2), also confirmed by ocular B ultrasonography. Finally, we revised the previous diagnosis to binocular VKH with secondary ACG, without extraocular signs of VKH. The laboratory results for uveitis included antinuclear antibody (ANA: +1:100 [granular type]) and anti-U1RNP antibody (positive). In addition, the laboratory results for HIV infection revealed that his CD4^+^ T-cell count was 296 cells/μl.

**Figure 3:**
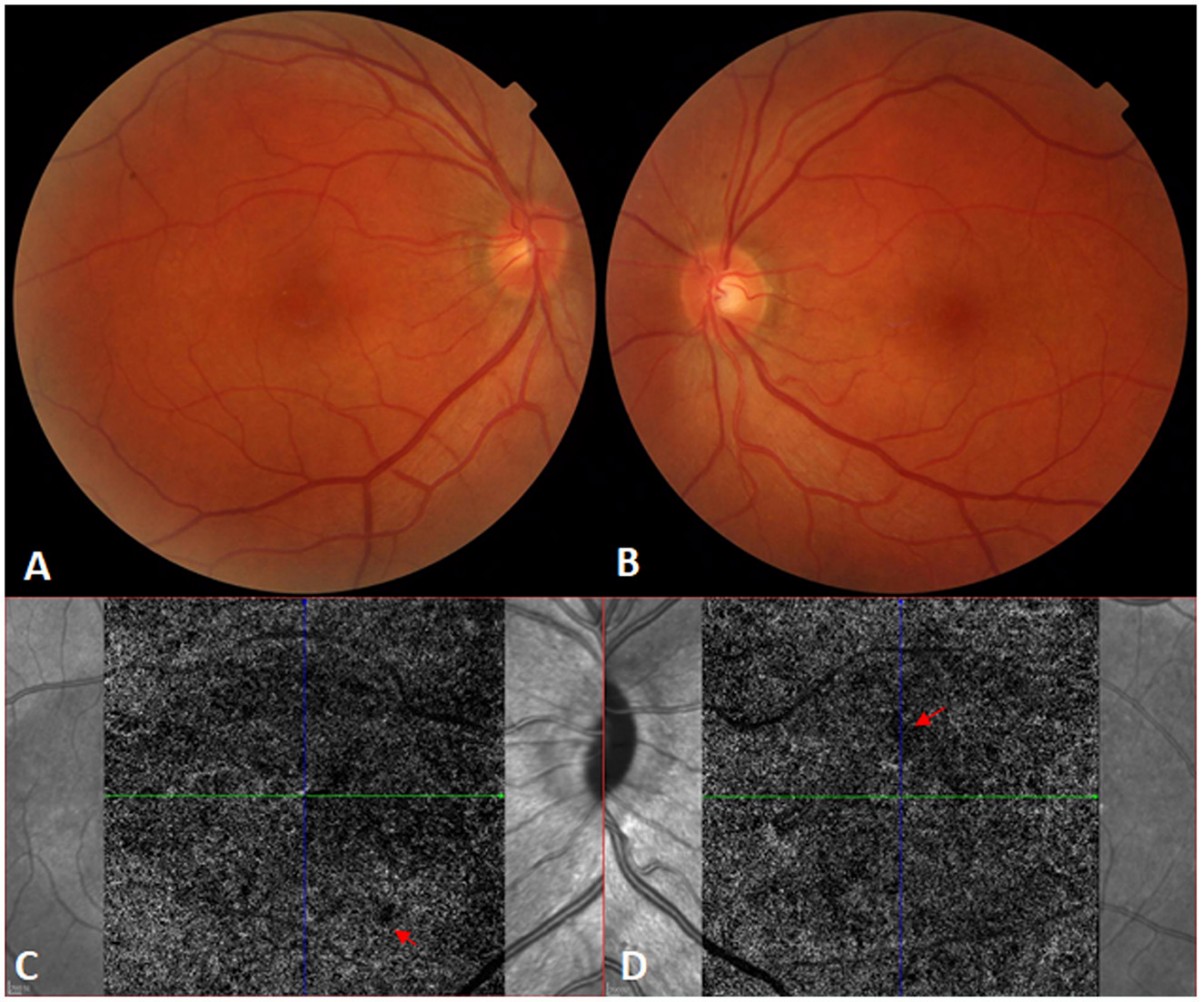
Recovery from Vogt-Koyanagi-Harada disease. **(A,B)** The fundus appears normal in the right and left eyes, respectively, after systemic corticosteroid therapy. **(C,D)** Optical coherence tomography angiography still shows bilateral dotted hypoperfusion of the choroidal capillaries [red arrows; **(C)**: right eye; **(D)**: left eye] 6 months after the onset of Vogt-Koyanagi-Harada disease.

After the administration of 105 mg prednisone once a day for 1 week, the IOPs of the right and left eye increased to 23 and 26 mmHg, respectively, with the aggravation of the serous retinal detachment (Figure 2). After continued corticosteroid therapy for 2 weeks, the patient's BCVA in both eyes improved to 1.0. The IOPs of the right and left eye were 16 and 14 mmHg, respectively. The multifocal serous retinal detachments resolved, and subretinal fluid (SRF) was completely absorbed in the OCT. The PACD regressed from about one-quarter corneal thickness to full (normal) thickness when examined by slit-lamp microscopy. Further treatment included 95 mg prednisone for 1 week, 85 mg prednisone for 1 week, 75 mg prednisone for 2 weeks, and 60 mg prednisone for 10 days, at which point oral prednisone was reduced by 5 mg per week. Six months after the onset of VKH, normal macular structures were obtained with the complete absorption of SRF (Figure 2). However, at this stage, dotted hypoperfusion of choroidal capillaries can still be observed in OCT angiography (Figure 3).

This study was reviewed and approved by the Institutional Review Board of the First Hospital of China Medical University, and written informed consent was obtained from the participant for the publication of this case report (including all data and images).

## Discussion

In this study, we present a rare case of secondary ACG involving VKH and HIV infection. Acute ACG as the first ocular manifestation of VKH is not common, and its pathogenesis may be acute inflammatory oedema, damage to the blood-aqueous humor barrier in the ciliary body, or the forward rotation of the iris and lens (7). In addition, HIV can also cause secondary glaucoma due to immune system damage and anti-optic nerve antibodies, anti-retinal antibodies, and cell lining proteins (α-fodrin) observed in the serum of HIV-infected patients (8, 9). Moreover, HIV-infected patients, who mainly have T-cell immune deficiency, can experience choroidal effusion, leading to secondary ACG with increased IOP (10). Kaushik et al. first reported a case of ACG secondary to tubercular choroidal granuloma with a mechanism similar to this case, including anterior rotation of the ciliary body at the scleral spur following the development of an inflammatory ciliochoroidal detachment (11).

To our knowledge, glucocorticoids are the first-line agents for the treatment of VKH. Glucocorticoid therapy administered during the acute phase slows down the chronic process and effectively prevents its onset in many patients. The combination of glucocorticoids and immunosuppressive drugs can help in both early vision recovery and the effective suppression of inflammation (12). In this study, unfortunately, the patient's IOP increased further after the initial systematic corticosteroid therapy, which may be partially due to the dominant hormone-sensitive genes (13). However, we did not assess the corresponding genes. In addition, glucocorticoids can inhibit phagocytosis of the trabecular cells, leading to a deposition of debris in the trabecular meshwork, blocking the outflow of aqueous humor, and increasing the IOP (14).

In this patient, the inflammation subsided after continued glucocorticoid treatment for 2 weeks, the acute inflammatory oedema of the ciliary body was reduced, the iris septum receded, and the anterior chamber angle opened (15). The serous macular detachment was aggravated after the initial corticosteroid therapy in this study. One possible explanation may be the presence of glucocorticoid and halocorticoid receptors in both retinal and choroidal vascular tissues; the combination of endogenous and exogenous glucocorticoids with the glucocorticoid receptor leads to an increase in the choroidal vascular dilation and permeability, increasing choroidal hydrostatic pressure (16). Hypoperfusion of choroidal capillaries after corticosteroid therapy also leads to the aggravation of retinal detachment (17). In this case, OCT angiography found dotted hypoperfusion of choroidal capillaries 6 months after the onset of VKH. Previously, we reported that in VKH cases, the vascular density of choriocapillaris increased significantly from 61.6 ± 2.2% at baseline to 65.1 ± 0.8, 65.7 ± 0.7, 66.1 ± 0.7, and 66.0 ± 2.0% at 1, 2, 3, and 6 months after glucocorticoid therapy, respectively (18).

Due to their anti-inflammatory and immunosuppressive effects, glucocorticoids can reduce the synthesis of inflammatory cytokines, inhibit the accumulation of inflammatory cells at inflammatory sites, reduce capillary permeability, and destroy the blood-retinal barrier, thereby reducing exudation, inhibiting tissue swelling, and reducing exudate retention, improving oedema, and promoting absorption of the SRF (19).

Increased autoimmunity and rheumatic diseases were observed in HIV-positive patients due to a disruption of the Th1/Th2 balance, stimulation of autoantibodies, chronic immune activation, immune confusion caused by HIV molecular mimicry, and increased cytotoxic T-cell response and immune dysregulation (20). The combined occurrence of VKH with HIV infection may be because they seem to act on similar cell lines (T lymphocytes) leading to immune dysfunction (21). Similarly, Graves' disease in HIV patients is associated with naive and primary thymic emigrant CD4+ T-cell recovery and inappropriate autoantibody production (22). In addition, the deterioration of Behçet's disease also has been associated with chronic HIV infection, and antiretroviral therapy for HIV will relieve the symptoms of Behçet's disease (20). Finally, posterior scleritis may develop several months after a significant rise in CD4+ T-lymphocytes, even after several years, in HIV patients (23).

This case study has some limitations; we missed information about the HIV status of the patient, including the viral load. In addition, we did not follow-up on the patient's HIV treatment to investigate its effect on the progress of VKH and secondary ACG. Finally, the UBM results from the follow-up were lost, and we had to use a slit-lamp microscope to evaluate the opening of the PACD.

In summary, this is the first report of secondary ACG in a patient with VKH and HIV infection. Both VKH and HIV infection contributed to the occurrence of ACG due to the leakage and forward rotation of the ciliary body and choroidal effusion. In clinical practice, it is important to understand the response of IOP and serous macular detachment after corticosteroid therapy.

## Data Availability Statement

The original contributions presented in the study are included in the article/supplementary material, further inquiries can be directed to the corresponding author/s.

## Ethics Statement

The studies involving human participants were reviewed and approved by the Institutional Review Board of the First Hospital of China Medical University. The patients/participants provided their written informed consent to participate in this study. Written informed consent was obtained from the individual(s) for the publication of any potentially identifiable images or data included in this article.

## Author Contributions

RH conceived of and designed the study, acquired the data, responsible for administrative, technical, material support, and supervised the study. RH and XB developed the methodology, analyzed and interpreted the data, wrote, reviewed, and revised the manuscript. All authors contributed to the article and approved the submitted version.

## Funding

This study was funded by the Beijing Bethune Charitable Foundation (No. AF-OG-03-1.1-03).

## Conflict of Interest

The authors declare that the research was conducted in the absence of any commercial or financial relationships that could be construed as a potential conflict of interest.

## Publisher's Note

All claims expressed in this article are solely those of the authors and do not necessarily represent those of their affiliated organizations, or those of the publisher, the editors and the reviewers. Any product that may be evaluated in this article, or claim that may be made by its manufacturer, is not guaranteed or endorsed by the publisher.
